# Concrete Properties Comparison When Substituting a 25% Cement with Slag from Different Provenances

**DOI:** 10.3390/ma11061029

**Published:** 2018-06-17

**Authors:** María Eugenia Parron-Rubio, Francisca Perez-García, Antonio Gonzalez-Herrera, María Dolores Rubio-Cintas

**Affiliations:** 1 Departamento de Ingeniería Industrial y Civil, Universidad de Cádiz, 11205 Algeciras, Spain; m.eugenia.parron@uca.es (M.E.P.-R.); mariadolores.rubio@uca.es (M.D.R.-C.); 2 Departamento de Ingeniería Civil, Materiales y Fabricación, Universidad de Málaga, 29071 Málaga, Spain; agh@uma.es (A.G.-H.)

**Keywords:** concrete, slag, valorization, cement, circular economy

## Abstract

Concrete consumption greatly exceeds the use of any other material in engineering. This is due to its good properties as a construction material and the availability of its components. Nevertheless, the present worldwide construction increases and the high-energy consumption for cement production means a high environmental impact. On the other hand, one of the main problems in the iron and steel industry is waste generation and byproducts that must be properly processed or reused to promote environmental sustainability. One of these byproducts is steel slag. The cement substitution with slag strategy achieves two goals: raw materials consumption reduction and waste management. In the present work, four different concrete mixtures are evaluated. The 25% cement substitution is carried out with different types of slag. Tests were made to evaluate the advantages and drawbacks of each mixture. Depending on the origin, characteristics, and treatment of the slag, the concrete properties changed. Certain mixtures provided proper concrete properties. Stainless steel slag produced a fluent mortar that reduced water consumption with a slight mechanical strength loss. Mixtures with ground granulated blast furnace slag properties are better than the reference concrete (without slag).

## 1. Introduction

Nowadays, the increasing growth of waste generated as a result of industrial activity is unavoidable, and dealing with this complex problem has become a difficult issue in part because of increasingly strict environmental regulations and policies. The analysis of industrial and construction waste and its transformation into raw materials in order to be introduced again into the production chain is part of the circular economy, a trend in economics initiated by Kenneth Boulding in 1965 [1].

According to the World Steel Association, 1600 millions of metric tons of steel were produced in the world in 2016, and around 162 million were produced in the European Union alone. Steel slag is a byproduct of steelmaking, produced during the separation of the molten steel from impurities in steelmaking furnaces. Making efforts to valorize this type of industrial waste can help achieve a more sustainable environment. Moreover, storing slags in landfills has negative effects on the environment, due to not only the extension of land occupied by this kind of waste, but also the leaching problems that it generates. According to the Nippon Slag Association, the pH of steel slag increases when reacting with water, which results in a highly alkaline fluid containing heavy metals that is harmful to the environment. This requires important and costly safety measures in landfills in order to verify the integrity of surrounding land and aquifers [2].

The properties of the slag produced by steelmaking depend on many factors, mainly the manufacturing process. According to Setie et al. [3], four types of steel slag can be distinguished: electric arc furnace (EAF) slag, blast furnace slag (BFS), basic oxygen furnace slag (BOFS), and ladle furnace slag (LFS).

Electric arc furnace slag is generated when iron scrap is melted and refined during the process of steelmaking. In a previous paper, Black Spanish EAF was characterized and was found to be composed of minerals such as anhydrous calcium silicates and silicoaluminates, magnetite and magnesioferrite, and manganese oxides (Luxán et al. [4]). Many researchers have observed the suitability of EAF as an aggregate substitution in concrete, showing similar compressive strength and high workability in concrete mixtures [5,6,7,8].

Regarding the specific case of steel slag waste valorization, in the European Union, ground granulated blast furnace slag (GGBF) has been used as an addition to the mix of cement manufactured in plant [9,10,11,12,13] to make up the cements CEM II and CEM III. This type of slag is a derivative of blast furnace slag, which is formed when iron ore or iron pellets, coke, and a flux (either limestone or dolomite) are melted together in a blast furnace. It has been used in various applications, mainly in aggressive environments (maritime and hydraulic facilities) because of its resistance against salt water and sulphates. Since the 80s, much research [14,15,16,17,18,19] has been conducted in order to use this type of slag as an additive or as fine and coarse aggregate substitutes [20,21,22,23,24,25,26,27,28,29].

Ladle furnace slag is produced during the secondary refining of steel through the addition of lime (CaO) and dolomitic limestone (CaO·MgO) in the electric arc furnace. The resulting byproduct, ladle furnace slag, has been used as a sand and cement substitute in masonry mortars, showing improvements in workability and mechanical strength in the medium term [30]. However, the use of LFS as a substitute in civil construction is less suitable due to its high expansiveness, especially when used in roads as an aggregate substitute [31,32,33,34].

During the manufacturing process of steel, 110–130 kg per metric ton of EAF and 20–30 kg per metric ton of LFS are generated [35].

In this paper, the suitability of three types of slag (EAF, GGBFS, and LFS) produced in still mills from different locations in Spain will be put to the test as cement substitutes in concrete mixtures.

A comparative analysis of different concrete mixtures will be carried out, substituting the cement with the aforementioned slags. The results of this paper are part of a wider program intended to elaborate a standard to guide the use of steel slag as a cement substitute in concrete mixtures in infrastructure building based on its chemical composition and mechanical behavior.

This investigation is an extension of the patent “Method for producing cinder concrete” [36], in which a manufacturing process for the production of concrete was developed with a stainless steel addition or cement substitution, obtaining a concrete mixture proportioning especially indicated for the construction of retaining walls and precast voussoirs, reducing energy and resources consumption.

## 2. Materials

Four different concrete mixes have been designed by substituting 25% of the weight of the cement with slag obtained from four different ladle furnaces in Spain. Because this investigation is part of a project studying the use of concrete with landfill slag substitution in precast voussoir manufacturing, the concrete dosage has been designed in order to meet certain consistency requirements (dry). The percent of the cement/slag substitution (25%) is based on the results of the patent “Method for producing cinder concrete” in order to make a performance comparison between the four types of slags. The chemical composition is shown in Table 1. The mix proportion shown in Table 2 was used as reference. The following materials were used to make the mixtures:

- Cement: Portland Cement CEM I 52.5 R with the composition given in Table 1. This cement was selected due to the absence of any kind of additive that could mask the results. It was used as reference pattern. Density: 2.5 g/cm^3^. Specific surface area: >2800 cm^2^/gr.
- Sand: crushed limestone sand was used. Size ratio: fine aggregate 0/2, medium aggregate (sand) 0/4, and gravel 4/16.
- Water: domestic tap water.
- Additive: Superplasticizer. Concrete additive: UNE EN 934-2.

The different slag used for the test were the following:

- Ground granulated blast furnace slag (GGBFS) with mechanical processing (M2): Initial aggregates are sand-like type 0/3 with a high humidity content (around 8–10%). They are dried and ground in origin. This is made by means of vertical roller mills specific to this material, which dries during grinding. This results in a maximum grain size of 0.063 mm; thus, it doesn’t require sieving. Density: 2.91 g/cm^3^. Specific surface area: 4620 cm^2^/gr.
- Unprocessed ladle furnace slag (LFS). Two different materials (with different compositions) were tested, coming from two different steel mills (used in M3 and M4, respectively). The only process they were subjected to was sieving in the lab with a 0.063 mm sieve. The fraction obtained through sieving was 23% and 15%, respectively.
- Unprocessed electric furnace slag from stainless steelmaking (M5), except sieving in the lab with a 0.063 mm sieve. The fraction obtained through sieving is 82%.

The chemical composition of the slags used in the concrete mixtures (M2, M3, M4, and M5) is shown in Table 1. This chemical composition is proportionated by the slag supplier companies, and it refers to the slags before the sieving process when needed in the laboratory. This provides more representative values for the different slags, because they do not depend on a particular batch of slag. However, chemical analysis of the slags that were introduced into the different concrete mixtures (after sieving when needed) were carried out in our laboratory and showed similar values to those given by the suppliers, without any remarkable deviation.

The different concrete mixtures were named as follow:

Mix 1 (M1): Ordinary concrete without slag.
Mix 2 (M2): Concrete with a 25% cement replaced with processed slag.
Mix 3 (M3): Concrete with a 25% cement replaced with unprocessed slag.
Mix 4 (M4): Concrete with a 25% cement replaced with unprocessed slag.
Mix 5 (M5): Concrete with a 25% cement replaced with stainless steel slag.

## 3. Tests Description

The different mixes described in the previous section were subject to different standard tests. The goal of these tests was to evaluate how the cement–slag substitution may affect the main properties of consistency and workability (slump test) and mechanical capabilities (compressive strength). Additionally, as one of the main uses of this kind of concrete is in the marine environment, the depth of penetration of water became a key characteristic to study.

Concrete was made with the mixture proportions shown in Table 2, where a 25% of the cement was substituted by the different slag according to Table 1, providing the five different mixes previously described.

The concrete mixture proportion was made according to the norm EN 12390-2 [37] for testing hardened concrete, where the making and curing of specimens for strength tests is described. It covers the preparation and filling of molds, the compaction of the concrete, the levelling of the surface, the curing of test specimens, and the transporting of the test specimens.

The compressive strength test specimen was a cube with a 10 cm size. Four concrete mixtures were made for each mix type. From each mixture, ten specimens were made; two were tested at 7, 28, and 90 days, leaving the others as reserve. The depth of penetration of water test was made with a cylindrical specimen with a 15 cm diameter and 30 cm height. Two different specimens were tested for every mix obtaining the average mean value.

### 3.1. Slump Test

The consistency of fresh concrete was determined by the slump test according to the norm EN 12350-2 [38]. A truncated conical mold was used where the fresh concrete was poured and compacted. The mold was placed over a base and was raised upwards. The concrete cone slumped and the distance slumped provided a measure of the consistency of the concrete. Table 3 presents the results of these tests.

This test evaluated the workability of the concrete. It was intended to evaluate how the cement–slag substitution altered the consistency, leading indirectly to change the water cement ratio or suggesting different uses.

### 3.2. Compressive Strength Test

**Figure 1:**
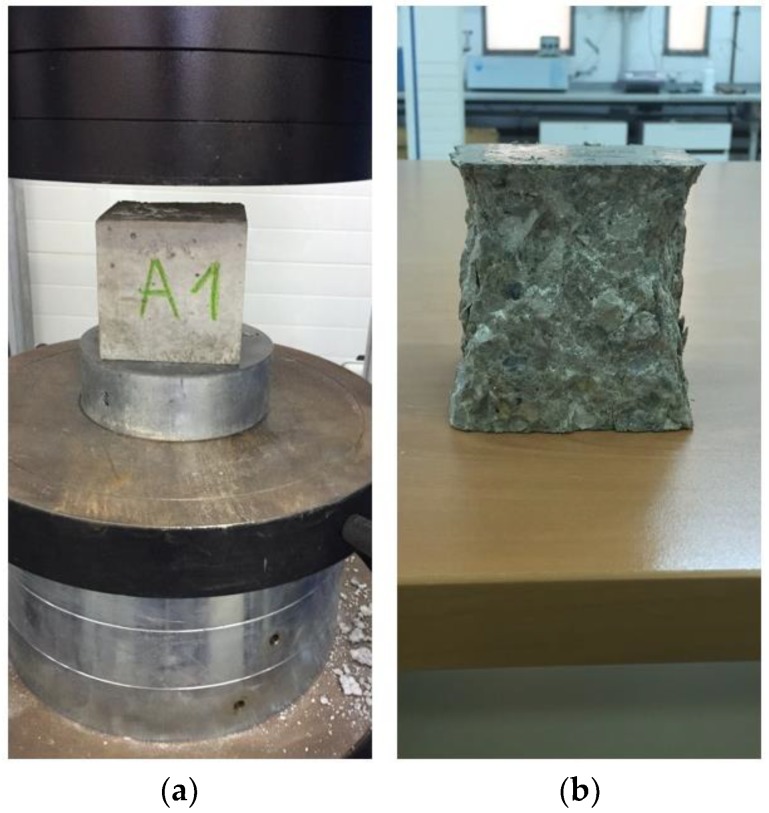
Compressive strength (**a**) test and (**b**) specimens.

Compressive strength tests were performed according to norms EN 12390-3 [39] and EN 12390-4 [40]. The test was carried out with a servo-controlled compact compression testing machine (Proeti, Madrid, Spain) with a maximum capacity of 2000 kN (ETIMATIC-Proetisa H0224). The load control system emulates a servo-valve using a pump that accurately controls oil flow to the piston, controlling the rotation speed of the pump motor and the load gradient. The superior compression plate is supported by a spherical bearing ring that accommodates the alignment inaccuracy and avoids any lateral force. Once the specimen is placed, the display continuously shows the load, the failure load, and the strength calculation in real time (Figure 1).

These tests provided the results shown in Table 4. The tests were performed at 7, 28, and 90 days to evaluate the behavior of slag in time. It was performed with a cubic specimen (10 cm size), and the load was applied at a constant speed of 0.5 MPa/s.

### 3.3. Depth of Penetration of Water Under Pressure Test

This test was made according to norm EN 12390-8 [41], depth of penetration of water under pressure, with a cylindrical specimen with a 15 cm diameter and 30 cm height.

The test was performed with a 28-days cured specimen. As a first step, the specimen was placed in a drying oven for 24–48 h to be completely dried.

Then, the specimen was placed in the apparatus and a water pressure of (500 ± 50) kPa was applied for (72 ± 2) h. The appearance of the surfaces was controlled during the test in order to detect any water leakage.

**Figure 2:**
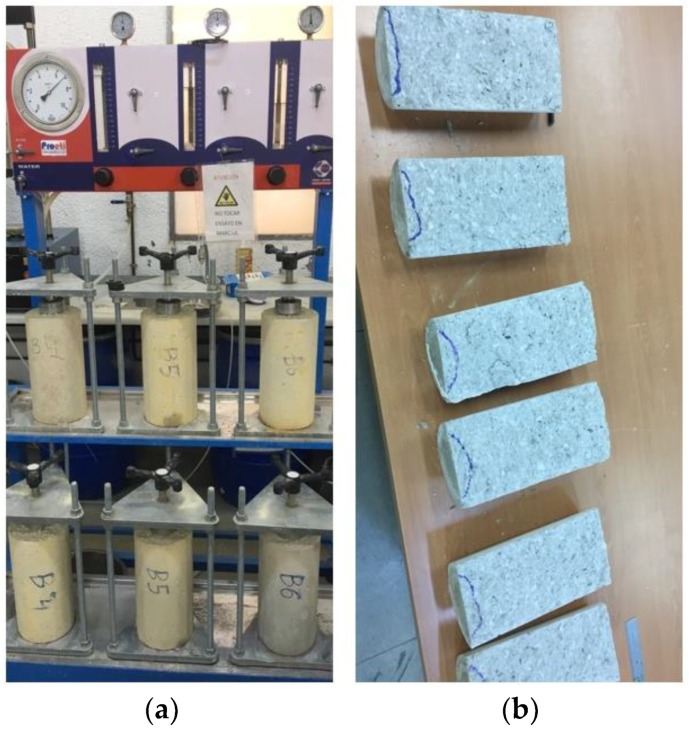
Depth of penetration of water under pressure (**a**) test and (**b**) specimens.

At the end of the test, the specimen was taken from the system and any excess of water was removed and wiped. Then, the specimen was broken in two halves perpendicularly to the face where the water pressure was applied. The water penetration front could be clearly seen and marked on the specimen (as seen in Figure 2). The maximum depth of penetration was measured in mm.

## 4. Results and Discussion

The results obtained with the different mixes shown in Section 2 are analyzed and discussed in this section.

### 4.1. Consistency

**Figure 3:**
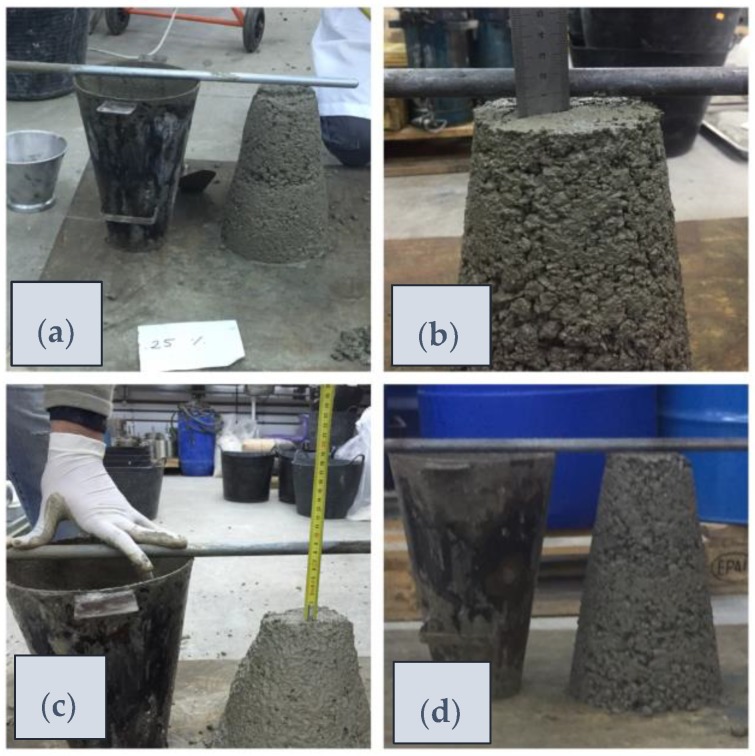
Concrete consistency. Slump test. (**a**) M1; (**b**) M2; (**c**) M5; and (**d**) M4.

The slump test results are shown in Table 3. The main observation is that most of the mixtures appear unaltered compared to the reference M1, except mixture M5 in which a higher fluency and workability could be observed (Figure 3c). It corresponded to the stainless-steel slag (M5), which was the only mix with a soft consistency. Consequently, the other mixtures showed a dry consistency, meeting the requirements for precast voussoir molds. A slump below half a centimeter was observed in the different tests. The material became very thick with a high presence of internal and external pores (Figure 3d). This behavior can be compared in Figure 3 with the other mixtures.

This means that the stainless-steel slag provoked a low level of friction in the material causing high fluency, as well as keeping the necessary viscosity to ensure the proper cohesion of the particles, thus avoiding segregation. This is the effect produced in self-compacting concrete [42], where fluency and viscosity is achieved by means of additives [43]. The opposite effect is observed in mix M4, where the material becomes denser and thicker, with a reduction in fluency and cohesion, promoting internal hollows and penalizing its permeability (as will be shown in the following subsections).

**Figure 4:**
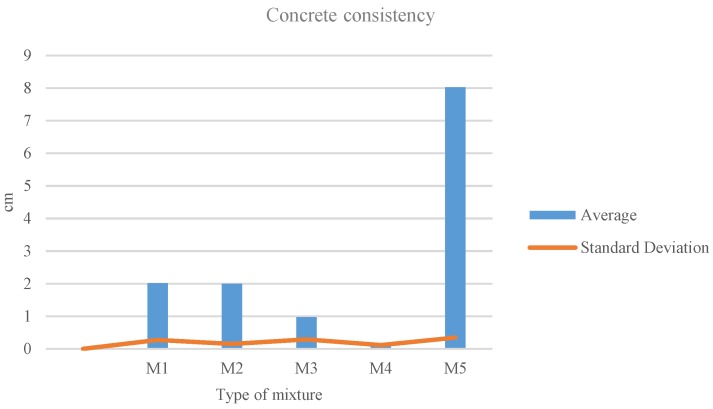
Consistency standard deviation.

Figure 4 shows the standard deviation values of the experiment. Average values are considered in the range from 0.5 to 2 cm. Three mixtures are present within this range, while M4 is below and M5 is very above.

### 4.2. Compressive Strength

**Figure 5:**
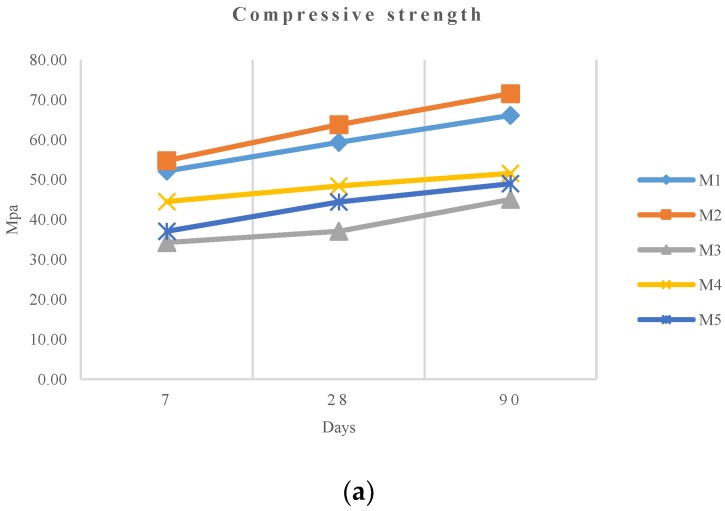

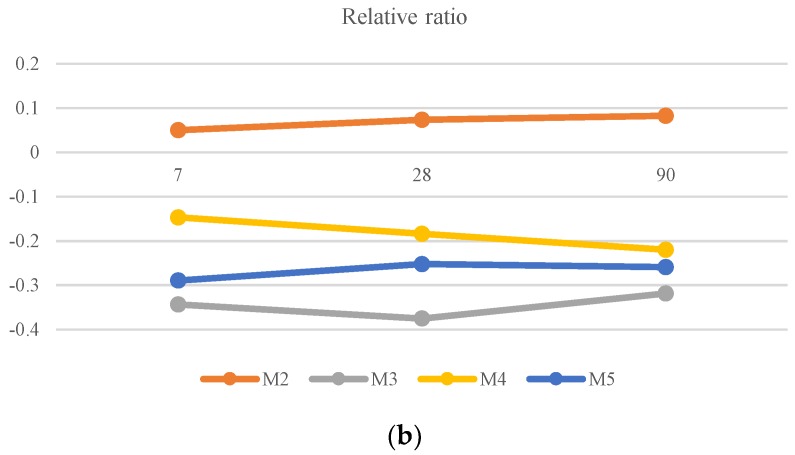
Compressive strength test results comparison: (**a**) evolution of different mixes over time and (**b**) relative ratio respect M1.

The compressive strength test results are shown in Table 4 and plotted in Figure 5, comparatively.

This experimental data shows how the M2 mixture provides a good compressive strength, even slightly above the reference sample, and 8% higher than that of M1. In Figure 5b, it can be observed how this relative compressive strength improvement is sustained over time. The other mixtures present certain strength loss, ranging from 32% for M3 to 22% for M4. This broad range—40% considering the four mixtures—proves that the origin, characteristics, and treatment given provide very different mechanical properties.

**Figure 6:**
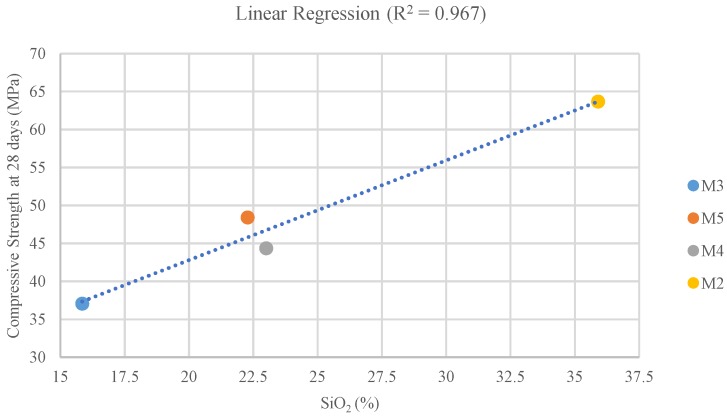
Pozzolanic capacity and compression strength correlation.

Regarding chemical composition, the proportion of SiO_2_ is the main factor to consider. Mix M4, with similar SiO_2_ content to the reference M1 (cement), shows the lower strength loss, and in the case of M2, where there is a strength gain, the SiO_2_ percentage is higher (Table 1). On the other hand, M2 and M5 with higher strength loss, have lower SiO_2_ content. In Figure 6, a linear regression is displayed showing the relationship between pozzolanic capacity and compressive strength. The correlation is strong with a R^2^ of 0.967.

According to Canovas et al. [44], pozzolanic materials with a high content of SiO_2_ present a high capacity to yield tobermorite (calcium hydrosilicates (C-S-H)) by reacting with portlandite (a product of concrete mineral hydration). This is the case of the M2 slag, which presents the highest SiO_2_ content, obtaining a superior compressive strength performance [45]. The slag used in M3, which provided the minimum compressive strength result at 28 days, presents the lowest percentage of SiO_2_. Thus, for the extreme cases (the highest and lowest SiO_2_ content) we can observe a direct effect of the pozzolanic capacity of the slag used in compressive strength performance. However, M4 slag and M5 slag, despite presenting similar SiO_2_ content (21–23%) to the cement, showed uneven results (48.42 and 44.38 MPa for M4 and M5, respectively, and 59.34 MPa for the 100% cement mix).

### 4.3. Depth of Penetration of Water

**Figure 7:**
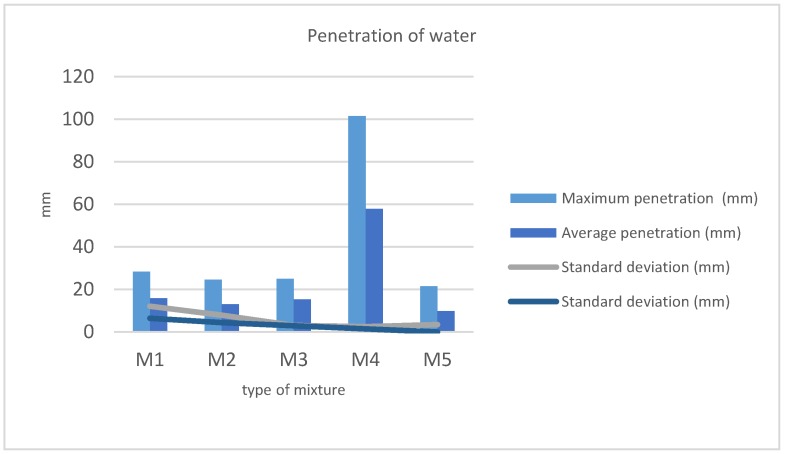
Average and standard deviation of the depth of penetration of water under pressure tests.

Depth of penetration of water under pressure test results are shown in Table 5 and plotted in Figure 7. Maximum and average penetration values are provided.

Excepting M4, all of the mixtures are in accordance with the norm EN 12390-8 [41]. The maximum deeper penetration is below the 30 mm limit established, and the average values do not exceed the 20 mm limit. M4 doubles both limits (Table 5). As seen in Section 4.1, it is the consequence of the appearance of internal cavities due to the very dry consistency of the concrete making a disperse aggregate mix, which cannot be eliminated by vibration. Water penetration is favored by this circumstance.

On the other extreme, we find the results obtained for the M5 mixture. In this case, penetration is below the average of the other samples. It is provoked by a reduction in the number and size of the cavities due to the good fluency and workability of this concrete.

The average values and the standard deviation of the experimental results are shown in Figure 7. M4 was not considered in the statistic calculation to avoid distortion.

Average values are considered in the range from 0.5 to 2 cm. Three mixtures are present within this range while M5 is below and M4 is very above.

## 5. Conclusions

According to the results described, we can outline the following conclusions:

(1) Regarding concrete consistency, stainless-steel slag furnaces (M5) provided excellent workability properties with a higher fluency, keeping a 0.5 water–cement ratio. On the other extreme, the M4 mixture provided a consistency that was extremely dry with cavities that undermined its properties. The other mixtures provided dry consistencies similar to that obtained with the reference mix (formulated with a low water–cement ratio).
(2) The ground granulated blast furnace slag (M2), with the highest content of SiO_2_, showed a compressive strength gain of 8% relative to concrete with no slag substitution. On the other hand, the slag with the lowest content of SiO_2_ performed the worst in the compressive strength tests, obtaining a strength loss of 32%. Thus, as has been pointed out by other authors [44,45], the content of SiO_2_ affects directly the resultant compressive strength of the concrete.
(3) Excepting the M4 mixture, the water penetration tests provided similar results for the different mixtures. In some cases, penetration was lower (M2 and M5) than the reference, and the reduction observed with M5 due to its exceptional fluency and workability was remarkable. M4 is the great exception and presents an extremely high maximum water penetration. This can be explained due to a possible lack of cohesion between particles during the curing process, because the consistency was too dry.

Summarizing, we can conclude that stainless-steel slag (M5) provides a good concrete formula, with excellent workability and a low water consumption. The negative point is a reduction in compressive strength. Regarding the type of slag, no correlation with compressive strength or workability has been found. It has been found that the results of the slag treated in a similar way to the cement (M2) are better than those obtained with the reference concrete (without slag), especially regarding the compressive strength, with the consequent savings in cement consumption.

As a final conclusion, we can say that cement substitution with slag can be considered a good strategy to reduce cement consumption while alleviating the waste management problem. Promising results have been obtained for certain mixtures, where the properties of the resultant concrete are even improved compared with the reference mix. This conclusion has been proven with 25% of cement substitution. Except for the pozzolanic capacity of the slag (SiO_2_ content), no strong correlation has been found to explain the resultant concrete properties. Ladle furnace slags (M3 and M4) showed dissimilar results for compressive strength and workability; thus, specific analysis must be carried out in order to assess the suitability of this type of steel waste to be valorized.

## Figures and Tables

**Table 1:** Cement and slag chemical composition (data provided by the supplying company).

Slag Origin/Chemical Composition	Type of Slag	SiO_2_	AL_2_O_3_	Fe_2_O_3_	CaO	MgO	Na_2_O	K_2_O	S	TiO_2_	Cl	Limestone	P_2_O_5_	Cr_2_O_3_	MnO	Fe
		%	%	%	%	%	%	%	%	%	%	%	%	%		
Cement (M1)	-	20–22	4–10	4	55–62	2	0.3	0.3	-	-	-	-	-	-	-	-
Processed slag (M2)	GGBFS	35.9	11.2	0.3	40	7.7	0.2	0.4	0.8	0.6	˂0.1	0.5	-	-	-	-
Unprocessed slag type 1 (M3)	LFS	15.85	16.53	0.83	57	7.7	-	-	1.46	-	-	-	˂0.1	˂0.1	0.53	-
Unprocessed slag type 2 (M4)	LFS	22.28	9.37	0.84	56.94	7.37	0	-	-	0.46	-	-	0	0	0.44	0.58
Stainless steel slag (M5)	EAF	23	5.27	1.41	56.9	6.23	-	-	-	1.5	-	-	˂0.1	2.96	1.68	-

**Table 2:** Concrete mixture proportion.

		Binder				Aggregates			
Mix	Water (*w*/*b* Ratio)	Dosage	Cement	Slag	Additive	Dosage	Fine Sand 0–2	Sand 0–4	Gravel 4–16
M1	0.5	300 kg/m^3^	100%	0%	3.9 kg/m^3^	2033.8 kg/m^3^	15%	35%	50%
M2-M3-M4-M5	0.5	300 kg/m^3^	75%	25%	3.9 kg/m^3^	2033.8 kg/m^3^	15%	35%	50%

**Table 3:** Slump test results.

Mix	Slump	Standard Deviation	Consistency
M1	2.0	0.3	Dry
M2	2.0	0.2	Dry
M3	1.0	0.3	Dry
M4	0.1	0.1	Dry
M5	8.0	0.3	Soft

**Table 4:** Compressive strength test results and standard deviation.

Days/Mixes	7	Standard Deviation (7)	28	Standard Deviation (28)	90	Standard Deviation (90)	% Strength Gain
M1	52.12	5.23	59.34	3.87	66.05	2.38	0%
M2	54.73	2.52	63.69	3.08	71.51	5.15	8%
M3	34.22	1.65	37.07	0.98	45.01	5.69	−32%
M4	44.48	1.02	48.42	0.34	51.54	0.54	−22%
M5	37.04	4.54	44.38	4.66	48.94	5.64	−26%

**Table 5:** Depth of penetration of water under pressure test results.

Mix	Maximum Penetration (mm)	Standard Deviation (mm)	Average Penetration (mm^2^)	Standard Deviation (mm^2^)
M1	28.3	12.09	15.8	6.42
M2	24.5	7.83	13.0	4.43
M3	25.0	3	15.3	2.91
M4	101.5	2.5	57.8	1.5
M5	21.5	3.5	9.8	0.015
